# Impact of Paliperidone Palmitate 6-Month Treatment After Hospital Discharge

**DOI:** 10.1192/j.eurpsy.2025.2092

**Published:** 2025-08-26

**Authors:** M. J. Mateos-Sexmero, O. Martin Santiago, B. Arribas-Simón, O. Segurado-Martín, C. Alario-Ruiz

**Affiliations:** 1 Psychiatry, Hospital Clínico, Valladolid, Spain

## Abstract

**Introduction:**

Rehospitalization is common in psychosis, often due to poor adherence to antipsychotic treatments. Long-acting injectable antipsychotics (LAIs), particularly paliperidone palmitate 6-month (PP6M), have shown promise in improving adherence and reducing relapses compared to monthly or quarterly formulations . Rapid initiation of PP6M during hospitalization may further optimize post-discharge outcomes and enhance the therapeutic adherence, minimizing the risk of a new outbreak, reducing the impact of rehospitalization and improving patients’ quality of life.

**Objectives:**

To evaluate clinical outcomes and treatment adherence in schizophrenia and other psychotic disorders after rapid PP6M initiation during psychiatric hospitalization.

**Methods:**

A retrospective analysis of 24 hospitalized patients diagnosded with schizophrenia and other psychotic disorders treated with PP6M within 7–10 days was conducted. Treatment adherence, follow-up attendance, and adverse effects were evaluated using McNemar’s test for statistical analysis.

**Results:**

Patients had a mean age of 36.8 years (SD=10.85), 64% were male, with an average of 2 prior hospitalizations (SD=3.16) in the past two years. Previously, 57% were on monthly LAIs. Post-discharge, 83% attended follow-ups. Antipsychotic monotherapy increased by 27% (p = .10) to 59%, while attendance at over 80% of appointments improved by 47% (p ≤ .001). Akathisia was reported in 25% of patients.

**Conclusions:**

PP6M significantly improves adherence by simplifying treatment regimens. Increased follow-up attendance (47%) and greater use of monotherapy reflect better patient outcomes. These findings align with prior evidence on the efficacy of LAIs in preventing relapses. Rapid initiation of PP6M can reduce rehospitalizations and optimize hospital resources. The low incidence of akathisia (25%) supports its safety and tolerability for long-term use.

**Disclosure of Interest:**

None Declared

